# Multi-Approach Analysis for the Identification of Proteases within Birch Pollen

**DOI:** 10.3390/ijms18071433

**Published:** 2017-07-04

**Authors:** Olivia E. McKenna, Gernot Posselt, Peter Briza, Peter Lackner, Armin O. Schmitt, Gabriele Gadermaier, Silja Wessler, Fatima Ferreira

**Affiliations:** 1 Department of Molecular Biology, University of Salzburg, Salzburg 5020, Austria; Oliviaeve.Mckenna@sbg.ac.at (O.E.M.); Gernot.Posselt@sbg.ac.at (G.P.); Peter.Briza@sbg.ac.at (P.B.); Peter.Lackner@sbg.ac.at (P.L.); Gabriele.Gadermaier@sbg.ac.at (G.G.); Silja.Wessler@sbg.ac.at (S.W.); 2 Department of Breeding Informatics, Georg August-Universität Göttingen, Göttingen 37073, Germany; armin.schmitt@uni-goettingen.de

**Keywords:** birch pollen, allergy, protease, proteome, transcriptome, zymogram

## Abstract

Birch pollen allergy is highly prevalent, with up to 100 million reported cases worldwide. Proteases in such allergen sources have been suggested to contribute to primary sensitisation and exacerbation of allergic disorders. Until now the protease content of *Betula verrucosa*, a birch species endemic to the northern hemisphere has not been studied in detail. Hence, we aim to identify and characterise pollen and bacteria-derived proteases found within birch pollen. The pollen transcriptome was constructed via de novo transcriptome sequencing and analysis of the proteome was achieved via mass spectrometry; a cross-comparison of the two databases was then performed. A total of 42 individual proteases were identified at the proteomic level. Further clustering of proteases into their distinct catalytic classes revealed serine, cysteine, aspartic, threonine, and metallo-proteases. Further to this, protease activity of the pollen was quantified using a fluorescently-labelled casein substrate protease assay, as 0.61 ng/mg of pollen. A large number of bacterial strains were isolated from freshly collected birch pollen and zymographic gels with gelatinase and casein, enabled visualisation of proteolytic activity of the pollen and the collected bacterial strains. We report the successful discovery of pollen and bacteria-derived proteases of *Betula verrucosa*.

## 1. Introduction

Ubiquitous to all living organisms, proteases function via cleavage of peptide bonds. All proteases act through stabilising the oxygen of the substrate peptide bond in an oxyanion hole, hence polarising the carbonyl group and making the carbon atom highly vulnerable to attack by an activated nucleophile [1]. Classifications of proteases are primarily based on the presence of molecules central to nucleophile formation, hence, deriving the four major catalytic classes of protease, i.e., cysteine, serine, metallo- and aspartic proteases [1,2]. Further detailed classification of proteases are described in the MEROPs database (Available online: http://merops.sanger.ac.uk). Each peptidase is provided an accession number correlating to the catalytic class (e.g., serine, cysteine). Accordingly, every peptidase belongs to a “family”, which represents a homologous group of proteins. Further classification into a “clan” defines evolutionary origins and can contain multiple families and occasionally multiple families of a distinct catalytic class [3].

The endogenous role of proteases in pollen is highly relevant in the process of germination; upon contact with a compatible stigma pollen grains hydrate, initiating a large release of solutes ultimately resulting in plant germination [4]. Proteases, some of which are situated on the exterior layers of pollen, itself, are included in this exudate and are released early in the hydration process [5,6]. Such pollen hydration is imitated upon contact with surface mucosa of the airway epithelium [5,7,8]. As a direct consequence, pollen-associated proteolytic activities have been implicated in the development of allergic diseases, with the capability to induce or enhance immune responses. An example includes the cysteine protease Amb a 11, derived from ragweed pollen, presenting both inducing and enhancing capabilities, as a sensitisation agent with proteolytic activity [9]. Other pollen-associated enzymes have been shown to compromise epithelial function, contributing to pollen allergenicity via the facilitation of allergens crossing such defensive barriers [10] or through direct activation of pattern recognition receptors [11]. Hence, pollen derived proteases pose a relevant source of investigation with relation to allergic sensitisation and development of allergic disease.

An excess of 100 million people worldwide have a reported allergy to birch pollen. Until now the protease content of *Betula verrucosa*, a species endemic to the northern hemisphere [12], has not been studied in detail. Hence, we aim to identify and characterise pollen and bacterial derived proteases found within *Betula verrucosa* pollen. We propose to employ a multi-approach analysis for the identification of proteases within commercial *Betula verrucosa* pollen extracts. Methods include proteomic and transcriptomic cross-referencing of the birch pollen and a protease assay to provide a quantitative measurement of proteolytic activity, with regards to the substrate casein. The application of freshly-collected birch pollen extracts to nutrient agar plates, together with Gram staining and 16S rRNA sequencing enabled identification of several bacterial isolates present in the pollen. For all samples, zymograms were performed for visual interpretation of the proteolytic activity.

## 2. Results

### 2.1. The Proteolytic Activity of Birch Pollen Extract

**Figure 1:**
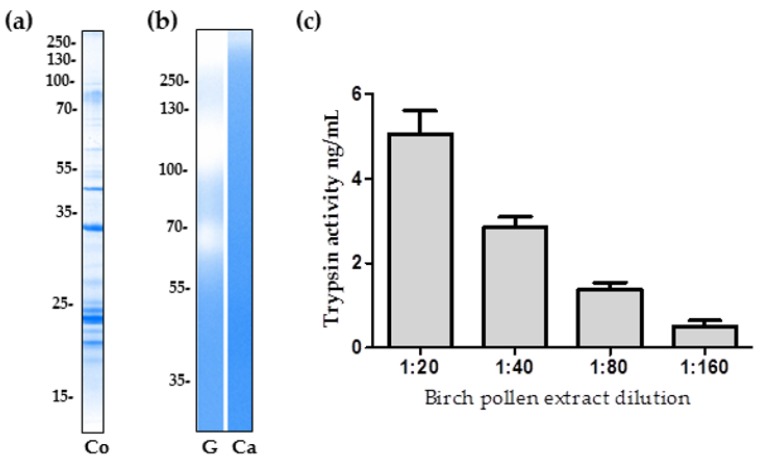
Protein separation and proteolytic activity of birch pollen extract. (**a**) Protein separation of 2 µg birch pollen extract was performed via SDS-PAGE under non-reducing conditions and stained with Coomassie Brilliant Blue (Co). The proteolytic activity of 5 µg of birch pollen extract was visualised via zymographic gels (**b**) using the substrates 0.1% gelatin (G) and 0.1% casein (Ca). Furthermore, a quantitative measure of the proteolytic content of birch pollen was determined using FITC-labelled casein using serial dilutions of the pollen extract as compared to a trypsin standard curve (**c**). Bars represent the mean values ± standard deviation (SD), for the three independent measurements.

To demonstrate the presence of proteins within the birch pollen extract (BPE), SDS-PAGE and Coomassie Brilliant Blue staining was performed (Figure 1a). The total protein concentration of the BPE, as determined by Bradford assay, was measured as 1.8 mg/mL. Gelatinase activity was shown in Figure 1b through the presence of three broad bands with a distribution of >250 kDa, 100–130 kDa, and ~70 kDa. Within the 0.1% casein gel, the visualisation of BPE caseinolytic activity is not as evident, with a faint band positioned above in the high molecular weight range (Figure 1c).

Using fluorescently labelled casein and, in reference to a trypsin standard curve, the protease activity of birch pollen was quantified as 0.61 ng/mg of pollen, with a standard deviation of ±0.09 ng/mg. A serial dilution of the BPE was performed and three independent experiments containing three replicates of each dilution were analysed (Figure 1c) and referenced to trypsin standard curves with *R*^2^ values between 0.95 and 0.98. The multiplication of each attained data point with the relevant dilution factor, when averaged, reveals the caseinolytic activity of the undiluted birch pollen extract in relation to trypsin activity, which translates to 0.61 ng/mg of birch pollen.

### 2.2. Transcriptomic and Proteomic Analysis

**Figure 2:**
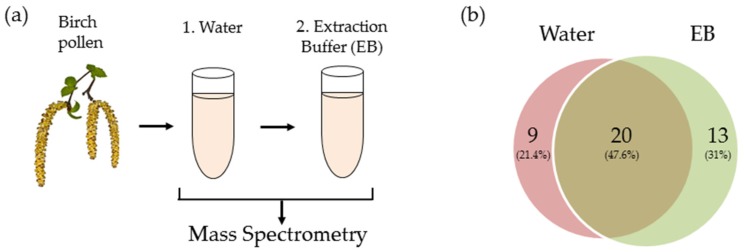
Sample preparation for mass spectrometry, consisted of two consecutive steps: (**a**) 1. Water extraction; 2. Trypsin extraction buffer (EB). The results of mass spectrometry analysis (cross-verified with transcriptomic data), identified a total of 42 proteases for both solutions, distributed as shown in (**b**). Birch pollen image adapted from image purchased from foltolia.com.

Transcriptomic analysis revealed proteases from 52 distinct families of the Pfam database. Data was referenced to the known sequence of *Arabidopsis thaliana*, a model organism widely used in molecular genetics. The NCBI sequence read archive (SRA) accession code for the RNA-sequencing reads of birch pollen is SRS2152558. The transcriptome expression levels for the proteases are provided in Höllbacher et al. [13]. Using a cross-referencing approach and with application to proteases alone, the proteome was compared to the transcriptome in order to identify birch pollen proteases in the extract. Two consecutive treatments (water and trypsin extraction buffer) were carried out on the BPE prior to mass spectrometry analysis and the exudates were analysed (Figure 2a). The collection of proteases obtained from the two different treatments potentially reflects differences in protein solubility. Following water extraction of the BPE, a total of 29 proteases were identified through proteomic and transcriptomic analysis (Figure 2b). Division into catalytic classes revealed serine (*n* = 8), cysteine (*n* = 7), aspartic (*n* = 4), metallo (*n* = 5), and threonine (*n* = 5)-type proteases.

**Figure 3:**
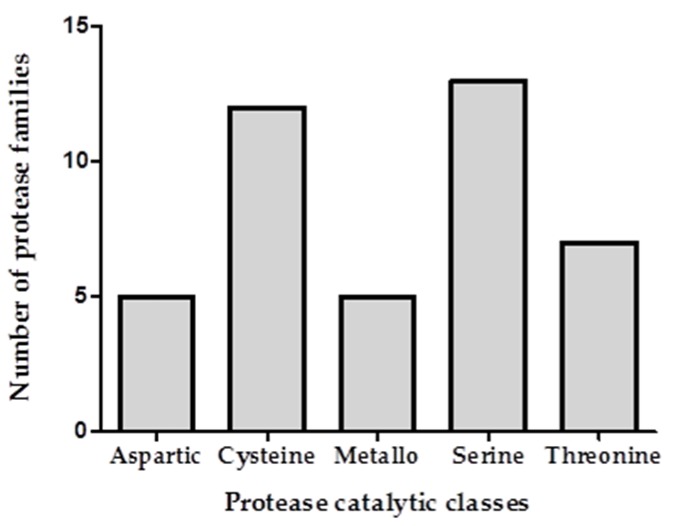
Total number of identified protease families for each identified catalytic class of proteases (aspartic, cysteine, metallo, serine, and threonine proteases).

Trypsin extraction buffer treatment of the BPE resulted in the identification of 33 proteases. The majority of which were identified as serine proteases (*n* = 11) along with cysteine (*n* = 9), threonine (*n* = 5) and metallo- (*n* = 5) proteases. An overlap of 20 proteases were identified between two different extractions. Combining the two datasets and removing duplicates revealed a total of 42 identified proteases (Table 1), the categorisation into catalytic classes is depicted in Figure 3. Water treatment resulted in nine distinct proteases, extraction buffer treatment revealed the identification of 13 morphologically distinct proteases. Between the two treatments an overlap of 20 proteases were identified. Within these 20 proteases one metallo- and two threonine proteases are not currently defined in the MEROPS database.

### 2.3. The Proteolytic Activity of Bacterial Isolates from Birch Pollen

**Figure 4:**
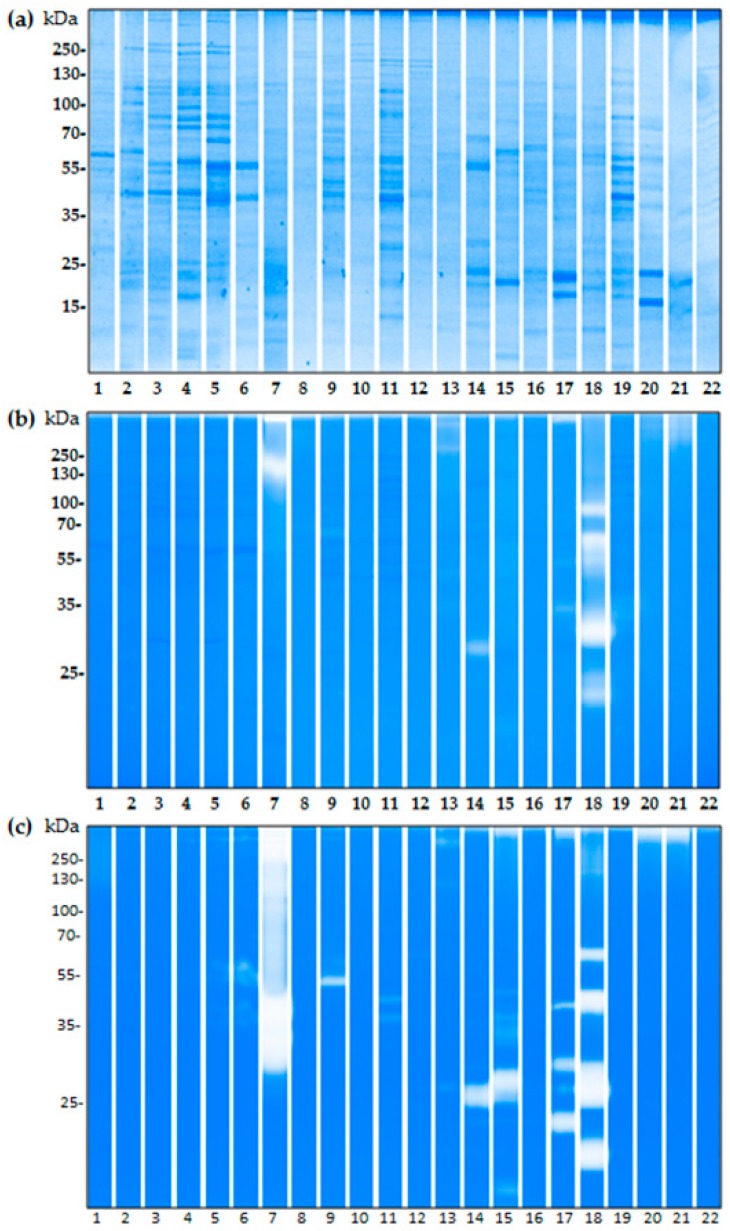
Gel electrophoresis was performed for (**a**) Coomassie stained SDS-PAGE gel and zymographic methods using (**b**) 0.1% casein (**c**) 0.1% gelatin for birch pollen derived bacterial isolates described by bacterial family in Table 2, numbered 1–22. (Gram negative: 1–7; Gram-positive: 8–22).

From the fresh pollen extracts we were able to successfully identify 22 bacterial isolates (seven Gram-negative and 15 Gram-positive) (Table 2) of which 17 strains presented with proteolytic activity as visually represented in either casein- or gelatin-based zymographic gels (Figure 4). For the casein substrate (Figure 4b), bacterial isolate number 7 of the Xanthomonadaceae bacterial family has two high molecular weight bands at 250 kDa and above. The isolate number 18 of the Bacillaceae family, is also present with strong proteolytic activity with at least five bands from below 25 to 100 kDa.

Within the casein gel (Figure 4b) the gram positive bacterial strains numbered 9, 13, 14, 15, 17, 21, show faint but existing proteolytic activity. Gelatinase activity is, in general, more prominent within the isolated bacterial strains, than that of casein. Up to ten of the identified bacterial strains present with gelatinase activity (Figure 4c). For isolates of the bacillaceae family numbered 15, 16, 18, and 19 a high gelatinase activity was observed. In particular, isolate number 8 of the Gordoniaceae family, presents a very saturated activity with over half of the lane stained. Sample 19, of the Bacillaceae family, shows at least four distinct bands, ranging from ~60 to 25 kDa. Lower activity is shown for Gram-positive samples 9, 12, 14, 21, 22. All of the bacterial isolates that present proteolytic activity within the gelatin substrate are Gram-positive.

## 3. Discussion

Whilst some proteases are, themselves, described as allergens [11], it has been heavily suggested that others play an assistive role in the development of allergy [14]. The respiratory epithelium consists of a complex set of epithelial cells which, in healthy individuals, maintains a finely-tuned homeostatic environment between internal and external stimuli, and provides a protective barrier against damaging external stimuli [15]. Proteases have been shown to disrupt the epithelial barrier integrity, through direct degradation of essential tight junction proteins [16] and via innate immune receptors involved in pro-inflammatory responses. In particular, protease activated receptor–2 (PAR-2) activation has been shown to alter the permeability between cells via p38 MAP kinase signalling [17]. Such disruption may promote inflammation, resulting in a primed environment for allergic sensitization to occur [18]. Further to this, the loss of epithelial integrity represents the ideal opportunity for allergens to enter and be detected by cells of the innate immune system [11]. In this context, we investigated the protease content of commercially available birch pollen. Initial zymogram experiments confirmed the presence of proteolytic activity in the birch pollen extracts for casein and gelatin substrates. To provide a quantitative measure of such activity we then performed a fluorescein isothiocyanate labelled casein protease assay. The assay revealed that birch pollen extract has a proteolytic activity of 0.61 ng per mg of pollen, with reference to the proteolytic activity of trypsin. Hence, we aimed to further identify the present proteases via a proteomic approach. The cross comparison of the proteome and transcriptome provided a database of proteases reliably identified to be of birch pollen origin. The analysis led to the successful identification of 42 proteases. The MEROPS database groups proteins of similar evolutionary origins into clans. Of the 54 described on MEROPS, our proteomic dataset describes proteases derived from 13 different clans. For further classification into a family of homologous proteases, our investigation revealed proteases deriving from 18 different families out of a total of 258 [19]. Of note are the five different families of cysteine proteases (C1, C2, C12, C19, and C85) deriving from the CA clan. Proteases of the C1 family (subfamily A) include papain [20], a highly relevant initiator of lung inflammation, as described using a mouse model, [21] and the well characterised proteolytic allergen of house dust mite, Der p 1 [22]. Homology to such proteases could be of significant relevance of understanding the process of allergic sensitization towards birch pollen. Hence, the further investigation of identified cysteine proteinases RD21A and RD19A, could be of high interest. The C2 family contains the calcium dependant enzyme, calpain. In particular, endogenous calpain-1 has been shown to contribute to IgE-mediated mast cell activation [23]. Furthermore, the involvement of calpain in eosinophilic disease is documented in detail [24]. In this context, calpain-type cysteine protease ADL1 also represents an interesting target for further investigation. With regard to their activity, metalloproteases, in particular those of the MA clan, are of relevance to allergy research. As their name suggests, metalloproteases perform their catalytic nucleophilic attack via a metal ion [25]. In particular, thermolysin, described as the prototypic protease of the MA clan, is able to elicit the activation of PAR-2 [26]. Our proteomic analysis recognized organellar oligopeptidase A (M3) and puromycin-sensitive aminopeptidase (M1) from the MA clan. Among all known proteases one third are classified as being serine proteases [27], which is reflective for our data set showing a total of 13 serine proteases of the total 42. Serine proteases are frequently referred to in the context of allergy, either as proteolytic allergens, e.g., Der f 6 [28] and Cur l 1 [29] or found within allergenic sources, for example in the highly-allergenic ragweed pollen [30,31]. In particular, the extracellular alkaline serine proteases and subtilisins, are highly relevant to allergy [32]. Cases of subtilisin allergy linked to the use of cleaning detergents containing bacteria-derived subtilisin enzymes have been reported. The discovery of a major allergen characterised as a subtilisin–like protease within the allergenic plant fungus, *Curvularia lunata* [33,34], is of interest to our data set with the identification of four subtilisin-like proteases, i.e., SBT1.7, SBT1.8, SBT4.15 and SBT5.4. It has been described in the literature that such proteases have the ability to cross-react and activate PAR-2 receptors [11,35]. The SC clan, family S10, consists primarily of serine carboxypeptidases; we have identified five such proteases, which share homology with the allergen Api m 9, derived from honey bee (*Apis mellifera*) [36]. Furthermore, the presence of environmental serine proteases has been linked to increased incidence of allergic disease [37], emphasizing the significance of further investigation.

Whilst endogenous birch pollen proteases are of key interest, we also wanted to investigate the potential proteolytic capability of the pollen microbiome. Recent studies exploring the microbiome highlight the potential significance of bacteria in allergen sensitisation [38]. A previous study, centred in Giessen, Germany, identified a complex birch pollen microbiome of 16 bacterial isolates [39]. We aimed to isolate bacteria derived from birch pollen within Salzburg, Austria, and tested for the presence of proteolytic activity. Our study has revealed a total of 22 bacterial isolates on the freshly-collected birch pollen, around half of which presented with proteolytic activity as visualised by gelatin and casein zymography. Eight of the 22 tested isolates belong to the Bacillaceae family which has been previously associated with allergy. In particular *Bacillus subtilis* of the Bacillaceae family, which excretes enzymes previously shown to elicit allergic responses [40]. Furthermore, the exploitation of their enzyme secreting capability in the detergent industry has posed a risk for occupational allergy and asthma [41,42]. Conversely, the application of *Bacillus subtilis* as an orally-administered probiotic [43] highlights the diverging outcomes, with regards to the development of allergy, of such protease exposure to differing physiological surfaces. Another well documented and highly relevant bacterial strain of the Bacillaceae family is *Lysinibacillus fusiformis*. The isolation of a serine metalloprotease from the bacterial strain *Lysinibacillus fusiformis* AU01 showed roles similar to that of the serine protease trypsin. The application of the yielded protease to adhering cell tissue cultures resulted in the dissociation of monolayers from tissue culture flasks suggesting the ability to disrupt cell–cell interactions [44], whilst the 16s rRNA sequencing did not. Our results show the presence of mild caseinolytic activity, as well as multiple bands of gelatinase activity. Further research is required to understand the impact such proteases have on epithelial cell surfaces.

Of the isolated bacterial strains, Gram-negative bacteria appeared to have less proteolytic power than Gram-positive strains. A difference in the transport mechanisms and the fact that most Gram-negative bacteria have pro-peptides, could provide one explanation as to the lack of activation present in the zymographic gels for Gram-negative bacteria, when compared to the positive strains [45]. In vitro/in vivo experimental conditions could provide a more relevant context, for bacterial protease release and future studies should focus on the in vivo relevance of such released proteases. Proteases are highly evolved; different classes of proteases are able to perform the same reaction via distinct mechanisms. Even within families of related proteases, the endogenous functions can show huge disparity, hence predicting the function of a protease presents as a difficult task [1,46]. Further to this, predictions of proteolytic activity based on homologous proteins, for example those in the same family/subfamily, could prove inaccurate, hence, further investigation into individual proteins is required for a full characterization. Moreover, the role proteases play in producing the phenotype of the pollen itself, is an interesting context in which to explore pollen and their associated enzymes. Our analysis has identified several highly relevant birch pollen associated proteases; the further study of which should contribute to the understanding of birch pollen sensitization and allergy development.

## 4. Materials and Methods

### 4.1. Pollen Extract Preparation

*Betula verrucosa* pollen (allergon AB, Thermo Fisher Scientific, Ängelholm, Sweden, Batch 012510101) prepared in water (180 mg/mL) was shaken for 12 h at 4 °C. Centrifugation at 13,000× *g* for 15 min at 4 °C enabled procurement of the supernatant. Protein concentration was then determined using Pierce Coomassie Brilliant Blue G-250 (Thermo Scientific, Vienna, Austria) according to manufacturer’s instructions. Using a spectrophotometer, absorbance was measured at 595 nm. 

### 4.2. Isolation of Bacteria on Pollen Grains

*Betula verrucosa* pollen was collected from five different trees in Salzburg, Austria. Ten milligrams of pollen was dissolved in 1 mL PBS. GC-agar plates (containing 5% FCS and 1 µg/mL Nystatin) and nutrient agar plates (containing 1 µg/mL Nystatin) were incubated at 20 °C and 37 °C together with 100 µL of the pollen suspension. Gram staining was performed on pure cultures of selected colonies and bacterial families were identified by 16S rRNA sequencing. Briefly, 16S RNA was amplified using 16S fwd (AGAGTTTGATCCTGGCTCAG) and 16S rev (AGGAGGTGATCCAACCGCA) primers. The resulting products had a size of approx. 1.5 kb and were subjected to DNA sequencing (MWG Eurofins, Ebersberg, Germany). Sequences were analysed using the NCBI-Basic Local Alignment Search Tool (BLAST; http://blast.ncbi.nlm.nih.gov 26.07.11) with the following parameters: Database: nr/nt; exclude uncultured/environmental sample sequences; megablast. Due to the high similarities in 16S sequences, the highest degree of qualitative analysis was the level of bacterial families. The selected bacteria were then cultured in nutrient broth and sonicated in lysis buffer (20 mM Tris pH 7.5, 1 mM EDTA, 100 mM NaCl, 1% Triton X-100, 0.5% DOC, 0.1% SDS, 0.5% NP-40). Samples were centrifuged for 15 min at 15,000× *g* to remove debris.

### 4.3. SDS-PAGE

Birch pollen extract and bacterial samples were mixed with 4× reducing sample buffer (125 mM Tris pH 6.8, 20% glycerol, 6% SDS, 0.02% bromophenol blue, 10% β-mercaptoethanol) and loaded with 15 µg of protein per well. Samples were heated for 5 min at 95 °C and run in 10% (*w*/*v*) polyacrylamide separation gels at 120 V for 150 min in SDS-Tris-glycine buffer, pH 8.05, and stained with Coomassie blue R-250 (Biorad, Munich, Germany).

### 4.4. Zymography

Ten percent (*w*/*v*) polyacrylamide gels were co-polymerised with either 0.1% type A gelatin from porcine skin (Sigma-Aldrich, Schnelldorf, Germany) or 0.1% casein from bovine milk (Sigma-Aldrich, Schnelldorf, Germany). Birch pollen extract and bacterial strains (with protein amounts of 5 µg for gelatin and 15 µg for casein per well) were added to 4× non-reducing sample buffer (125 mM Tris pH 6.8, 20% glycerol, 6% SDS, 0.02% bromophenol blue). Samples were incubated at 25 °C for 5 min before being loaded into gels. Following electrophoresis at constant 100 V at room temperature. Gels were incubated two times for 30 min in wash buffer (2.5% Triton X-100 in H_2_O) solution at room temperature, then incubated at 37 °C for 16 h in developing buffer (10 mM Tris pH 7.5 with 5 mM CaCl_2_, 1 µM ZnCl_2_). Thereafter, gels were stained with 30% (*v*/*v*) methanol and 10% (*v*/*v*) acetic acid, containing 0.5% (*w*/*v*) Coomassie brilliant blue R-250 (Biorad, Vienna, Austria). De-staining was performed with 50% (*v*/*v*) methanol and 10% (*v*/*v*) acetic acid. Gelatinase activity was visualized as unstained bands on a blue background, representing areas of proteolysis of the substrate protein. 

### 4.5. Protease Activity Assay

Quantification of birch pollen proteolytic activity was performed using a fluorescein isothiocyanate labelled casein protease assay kit (Pierce, Thermo Scientific, Vienna, Austria). Trypsin was used as a standard, ranging from 0.008 to 0.5 µg/mL. Birch pollen extract was prepared freshly in a two-fold serial dilution and CaCl_2_ was added to a final concentration of 100 µM. The measurements were performed in a white flat bottom 96-well plate (Nunc, Roskilde, Denmark). The fluorescence was measured in a Tecan Infinite 200 PRO (Tecan Group Ltd., Männedorf, Switzerland) plate reader with the filter setting 485/535 nm (Ex/Em). Three independent experiments containing three replicates of each dilution were analysed. The mean and standard deviation of the data points were calculated. 

### 4.6. RNA Sequencing

Total RNA was isolated from the pollen of *Betula verrucosa* (allergon AB, Thermo Fisher Scientific, Ängelholm, Sweden, batch 012510101). Five hundred milligrams of pollen were homogenised and resuspended in 4.2 M of guanidine thiocyanate, 50 mM of BES pH 7.2, 4 mM Ethylenediaminetetraacetic acid (EDTA), supplemented with 1% (*v*/*v*) β-mercaptoethanol, 1% (*w*/*v*) *N*-lauryl-sarkosyl, and 1% (*v*/*v*) n-butanol. Following centrifugation at 15,000× *g* for 15 min, RNA was extracted from the supernatant using TRIzol LS reagent (Invitrogen, Carlsbad, CA, USA), according to the manufacturers’ manual.

RNA-sequencing was performed with Illumina’s HiSeq 2500 system and delivered 220 m paired reads. Per base sequence quality was encoded in the Phred +33 format and assessed using FastQC: A quality control tool for high throughput sequence data (Avaialbe online: http://www.bioinformatics.babraham.ac.uk/projects/fastqc). Using Trimmomatic [47], low quality bases from the ends of reads were removed and pairs where both reads passed the checkpoint were processed. As the full genome for *Betula verrucosa* is not currently available, the Trinity [48] pipeline was used to sequentially apply the three tools; Inchworm, Chrysalis, and Butterfly for de novo transcriptome assembly. Quantification of the assembly was performed with Kallisto.

Quality parameters and general statistics were obtained by FastQC, the fastqutils from NGSUtils [48] and from the RNA-Seq provider’s report. The transcripts were then functionally annotated using blastx and UniProt/SwissProt (version 11_2016). As databases. The e-value cutoff for blastx was set to 0.001. The NCBI SRA accession code for the RNA-seq reads is SRX2769122. The expression levels for the proteases are given in Höllbacher et al. [13].

### 4.7. Mass Spectrometry Analysis

For mass spectrometric analysis, the extracts were digested with the ProteoExtract All-in-One Tryps For mass spectrometric analysis, the extracts were digested with the ProteoExtract All-in-One Trypsin Digestion Kit (EMD Millipore, Billerica, MA, USA). Resulting peptides were desalted using C18 ZipTips (EMD Millipore) and separated by reverse-phase nano-HPLC (Dionex Ultimate 3000, Thermo Fisher Scientific, Bremen, Germany, column: PepSwift Monolithic Nano Column, 100 µm × 25 cm, Dionex). The column was eluted with an acetonitrile gradient (Solvent A: 0.1% (*v*/*v*) FA/0.01% (*v*/*v*) TFA/5% (*v*/*v*) DMSO; solvent B: 0.1% (*v*/*v*) FA/0.01% (*v*/*v*) TFA/90% (*v*/*v*) ACN/5% (*v*/*v*) DMSO; 5–45% B in 60 min) at a flow rate of 0.8 µL/min at 55 °C. Peptides were analysed with a Q Exactive Orbitrap mass spectrometer (Thermo Fisher Scientific, Bremen, Germany,) directly coupled to the HPLC. Capillary voltage at the nano electrospray head was 2 kV, the instrument was tuned for maximum sensitivity. For peptide assignments, a top 12 method was used with a normalized fragmentation energy at 27%. Protein assignment was done with PEAKS Studio 8 (Bioinformatics Solutions, Waterloo, Canada). As a sequence database we took the annotated transcripts as described above, and translated to all six protein sequence reading frame. Only peptide hits with a probability score (−10logP) ≥ 35 were used for protein identification.

We took the annotated transcripts as described above, and translated to all six protein sequence reading frames.

### 4.8. Further Analysis

Analysis of the cross referenced proteome/transcriptome data was carried out manually.

## Figures and Tables

**Table 1:** Proteases identified via proteome and transcriptome cross-analysis of commercial birch pollen extracts.

Catalytic Type	Description	MEROPS Accession	Clan (Subclan)	Family (Subfamily)	Ext.
Aspartic	Aspartic proteinase A1	A01.A02	AA	A1 (A)	EB, W
Aspartic	Aspartic proteinase CDR1	A01.069	AA	A1 (B)	EB, W
Aspartic	Aspartic proteinase oryzasin-1	A01.020	AA	A1 (A)	EB
Aspartic	β-secretase 2	A01.041	AA	A1 (A)	W
Aspartic	Signal peptide peptidase	A22.A15	AD	A22 (B)	W
Cysteine	Calpain-type cysteine protease ADL1	C02.019	CA	C2 (A)	EB
Cysteine	Cysteine protease RD19A	C01.022	CA	C1 (A)	W
Cysteine	Cysteine proteinase RD21A	C01.064	CA	C1 (A)	EB, W
Cysteine	Desumoylating isopeptidase 1	C97.001	CP	C97	EB
Cysteine	OTU domain-containing protein 5	C85.001	CA	C85 (B)	EB
Cysteine	Protein DJ-1 homolog D	C56.A01	PC (C)	C56	EB, W
Cysteine	Thiol protease aleurain-like	C01.162	CA	C19 (A)	W
Cysteine	Ubiquitin carboxyl-terminal hydrolase	C12.A03	CA	C12	EB, W
Cysteine	Ubiquitin carboxyl-terminal hydrolase	C19.094	CA	C19	EB, W
Cysteine	Ubiquitin carboxyl-terminal hydrolase	C19.068	CA	C19	W
Cysteine	Ubiquitin thioesterase OTU1	C85.007	CA	C85 (B)	EB
Cysteine	Ubiquitin-like-specific protease 1D	C48.A04	CE	C48	EB
Metallo	Leucine aminopeptidase 2	n/a	MF	M17	EB, W
Metallo	Mitochondrial-peptidase subunit α 2	n/a	n/a	n/a	EB, W
Metallo	Organellar oligopeptidase A	M03.A01	MA (E)	M3 (A)	EB, W
Metallo	Probable Xaa-Pro aminopeptidase P	M24.A10	MG	M24 (B)	EB, W
Metallo	Puromycin-sensitive aminopeptidase	M01.029	MA (E)	M1	EB, W
Serine	ATP-dependent Clp protease subunit 5	S14.A01	SK	S14	EB
Serine	Dipeptidyl peptidase family member 6	S09.A77	SC	S9	EB, W
Serine	Probable glutamyl endopeptidase,	S09.021	SC	S9 (D)	EB
Serine	Serine carboxypeptidase-like 20	S10.A11	SC	S10	EB, W
Serine	Serine carboxypeptidase-like 40	S10.A41	SC	S10	W
Serine	Serine carboxypeptidase-like 42	S10.A21	SC	S10	W
Serine	Serine carboxypeptidase-like 48	S10.A46	SC	S10	EB, W
Serine	Serine carboxypeptidase-like 49	S10.A45	SC	S10	EB, W
Serine	Subtilisin-like protease SBT1.7	S08.112	SB	S8 (A)	EB, W
Serine	Subtilisin-like protease SBT1.8	S08.A24	SB	S8 (A)	EB
Serine	Subtilisin-like protease SBT4.15	S08.A13	SB	S8 (A)	EB
Serine	Subtilisin-like protease SBT5.4	S08.A26	SB	S8 (A)	EB, W
Serine	Tripeptidyl-peptidase 2	S08.A56	SB	S8 (A)	EB
Threonine	Proteasome subunit α type-3	n/a	n/a	n/a	W
Threonine	Proteasome subunit α type-5-B	T01.995	PB (T)	T1 (A)	EB, W
Threonine	Proteasome subunit α type-6	T01.971	PB (T)	T1 (A)	EB, W
Threonine	Proteasome subunit α type-6-B	n/a	n/a	n/a	EB
Threonine	Proteasome subunit α type-7	n/a	PB (T)	T1 (A)	EB, W
Threonine	Proteasome subunit β type-4	T01.987	PB (T)	T1 (X)	W
Threonine	Proteasome subunit β type-5-B	T01.A10	PB (T)	T1 (A)	EB

Abbreviations: Ext. = Extraction method, EB = extraction buffer (trypsin based), W = water, n/a = not available.

**Table 2:** Identified bacterial strains from freshly collected birch pollen.

Gram Stain	No. in Gel	Bacterial Order	Bacterial Family	C	G
negative	1	Caulobacterales	Caulobacteraceae	–	+
	2	Enterobacteriales	Noctuoideaceae	–	–
	3	Pseudomonadales	Pseudomonadaceae	–	–
	4	Pseudomonadales	Pseudomonadaceae	–	–
	5	Pseudomonadales	Pseudomonadaceae	–	–
	6	Sphingomonadales	Sphingomonadaceae	–	+
	7	Xanthomonadales	Xanthomonadaceae	++	+++
positive	8	Actinomycetales	Gordoniaceae	–	–
	9	Actinomycetales	Microbacteriaceae	+	+
	10	Actinomycetales	Micrococcaceae	–	–
	11	Actinomycetales	Micrococcaceae	–	+
	12	Actinomycetales	Nocardioidaceae	–	–
	13	Actinomycetales	Streptomycetaceae	+	+
	14	Bacillales	Bacillaceae	+	++
	15	Bacillales	Bacillaceae	–	++
	16	Bacillales	Bacillaceae	–	–
	17	Bacillales	Bacillaceae	+	++
	18	Bacillales	Bacillaceae	++	+++
	19	Bacillales	Bacillaceae	+	–
	20	Bacillales	Bacillaceae	–	+
	21	Bacillales	Bacillaceae	+	+
	22	Bacillales	Paenibacillaceae	–	–

Abbreviations: C = casein, G = gelatin, (proteolytic activity ranging from high = +++ to none = –).
